# Inequalities in non-small cell lung cancer treatment and mortality

**DOI:** 10.1136/jech-2014-205309

**Published:** 2015-06-05

**Authors:** Ula Nur, Manuela Quaresma, Bianca De Stavola, Michael Peake, Bernard Rachet

**Affiliations:** 1 CRUK Cancer Survival Group, Department of Non-communiicable Disease Epidemiology, London School of Hygiene and Tropical Medicine, London, UK; 2 Department of Medical Statistics, London School of Hygiene and Tropical Medicine, London, UK; 3 National Cancer Intelligence Network, Public Health England, London, UK

**Keywords:** CANCER, Epidemiology of chronic non communicable diseases, MORTALITY

## Abstract

**Background:**

Non-small cell lung cancer (NSCLC) comprises approximately 85% of all lung cancer cases, and surgery is the preferred treatment for patients. The National Health Service established Primary Care Trusts (PCTs) in 2002 to manage local health needs. We investigate whether PCTs with a lower uptake of surgical treatment are those with above-average mortality 1 year after diagnosis. The applied methods can be used to monitor the performance of any administrative bodies responsible for the management of patients with cancer.

**Methods:**

All adults diagnosed with NSCLC lung cancer during 1998–2006 in England were identified. We fitted mixed effect logistic models to predict surgical treatment within 6 months after diagnosis, and mortality within 1 year of diagnosis.

**Results:**

Around 10% of the NCSLC patients received curative surgery. Older deprived patients and those who did not receive surgery had much higher odds of death 1 year after being diagnosed with cancer. In total, 69% of the PCTs were below the lower control limit of surgery and have predicted random intercepts above the mean value of zero of the random effect for mortality, whereas 40% were above the upper control limit of mortality within 1 year.

**Conclusions:**

Our main results suggest the presence of clear geographical variation in the use of surgical treatment of NSCLC and mortality. Mixed-effects models combined with the funnel plot approach were useful for assessing the performance of PCTs that were above average in mortality and below average in surgery.

## Introduction

Lung cancer is the most common cancer diagnosed in the world and is responsible for a quarter of male cancer deaths and one-fifth of female cancer deaths. A total of 1.8 million cases were diagnosed in 2012, accounting for around 13% of all new cancer cases.^1^ In 2011 in the UK, lung cancer was the second most common cancer in men (23 770 new cases) and women (19 693 new cases), accounting for 14% of all cancer cases in men and 11% in women.^2^ There has been a steady decline in the number of incidence cases in men and a modest increase in the number of cases in women in the past 30 years in the UK and many other countries worldwide.

Lung cancer is divided into two main groups: small cell lung cancer (SCLC) and non-small cell lung cancer (NSCLC). The latter comprises approximately 85% of all lung cancer cases. The most common types of NSCLCs are adenocarcinoma, squamous cell carcinoma and large cell carcinoma. Surgery is the preferred treatment for patients with early-stage NSCLC providing the greatest chance of long-term survival in such patients. The overall proportion of all patients with lung cancer undergoing surgical resection in England between 2004 and 2006 was less than 10%, with older patients having the lowest likelihood of resection,^3^ though the number of resections has been increasing in recent years.^4^ A UK hospital recently reported a resection rate of 25%, which is comparable with the European standard,^5^ and the annual report of the English National Lung Cancer Audit (NLCA) reporting data on patients first diagnosed in 2012 (National Lung Cancer Audit 2013) reported a resection rate of 22% in patients with biopsy-confirmed NSCLC. However, in that report, the NLCA also demonstrated that even in patients with stages I and II NSCLCs, the resection rate varied by region (Cancer Networks) from 32% to 65%. Surgical treatment in patients with NSCLC decreases considerably with the age of the patient at diagnosis,^4^ ^6^ while outcomes of treatment of patients diagnosed at an older age are very similar to those of younger patients.^7^

Survival from lung cancer has remained poor in England, and although there have been recent improvements in 1-year survival rates,^8^ ^9^ there has only been very slight improvement in 5-year survival in the past decade;^10^ 5-year survival was found to be among the lowest of six worldwide countries with comparable wealth.^11^ Persistent geographic inequalities in cancer survival are also seen within the UK, with a clear north-south gradient when survival between Primary Care Trusts (PCTs) was compared over time^12^ ^13^ and for cancer networks.^14^ ^15^

The National Health Service (NHS) Cancer Plan^16^ set out a national programme, the aim of which was to improve cancer outcomes by improving early diagnosis, screening and access to treatment for all patients with cancer. Extra funding was released, with the actual expenditure on cancer reaching £636 million in 2003–2004. The NHS established PCTs in 2002 to manage local health needs, subject to national standards and guidance. In April 2013, the 151 PCTs that were responsible for the management of 80% of the NHS budget were replaced with clinical commissioning groups (CCGs) numbering 211 at the time of writing. The health-related outcome of patients living in the same geographical area which falls under the management of the same PCT may be more similar to each other than those of patients from different areas. They are more likely to share similar social and lifestyle characteristics and healthcare facilities. There was also concern at that time that not all of the funding allocated by the NHS for cancer was in fact spent on cancer services, and therefore in 2004 PCTs were asked to report the proportion of their budget spent on major diseases such as cancer. The King's Fund released a report in 2006 showing significant variation in budgets for cancer reported by PCTs.^17^

Socioeconomic disparities have been shown in lung cancer survival for both sexes, with lower survival among deprived groups.^18–20^ A study of the survival of patients with lung cancer in Scotland found that although deprived patients had the same chance of receiving curative treatment, they were less likely to survive 3 years after diagnosis than affluent patients.^21^ Inequalities were also observed in access to treatment and survival in 26 health authorities in South East England.^22^ Higher overall treatment rates in the Yorkshire region of England have been shown to be associated with better survival rates.^23^

The main aim of this study is to investigate whether PCTs with a lower uptake of surgical treatment are those with above-average mortality 1 year after diagnosis, and to assess the impact of known factors influencing these two outcomes, such as age at diagnosis, socioeconomic deprivation, sex and PCT spending on lung cancer. With continuous change in the size and geographical boundaries of administrative bodies responsible for the management, planning and funding of healthcare in England, we believe that the methods described here can be used to monitor and compare the performance and outcomes of all administrative bodies, irrespective of their size and number.

## Methods

### Data

All adults within the age range 15–99 years diagnosed with a first primary, invasive lung cancer (ICD 10 C33-C34) in 1998–2006 and registered in one of the eight regional English cancer registries were identified. The Office for National Statistics provides information on each patient's vital status (alive, dead, emigrated or lost to follow-up) and their postcode of residence at diagnosis, from which patients were assigned to one of five deprivation categories (from 1 most affluent to 5 most deprived) using their postcode of residence at diagnosis. Only ecological measures of deprivation derived from the socioeconomic characteristics of the Lower Super-Output Area (LSOA) in which each patient was resident at the time of diagnosis, but not individual-level information, were available. Deprivation categories were defined from the income domain score of the IMD (2004)^24^ using administrative data for the 34 378 LSOAs in England.

Each cancer record was linked to an extract of the Hospital Episode Statistics (HES)^25^ database from 1998 to 2006 using NHS number, sex, date of birth and postcode at time of diagnosis. Cancer registry data were available for patients diagnosed up to the end of 2009 and followed up to the end of 2010. Data for HES episodes were only available up to the end of 2006, and therefore it was decided to restrict the analysis to the patients diagnosed in 1998–2006. The first curative surgical procedure was identified from each linked patient record and was considered for analysis if it included OPCS-4 episode procedure codes (Office of Population Census and Surveys Classification of Surgical Operations and Procedures-4) of excision of lesion of trachea (E391, E398, E399), excision of carina (E441), resection of bronchus and anastomosis (E461), total pneumonectomy (E541), bilobectomy (E542), lobectomy (E543), excision of lung segment (E544), partial lobectomy (E545), excision of lung (E548) E549), excision of lung unspecified (E549), excision of lesion of lung (E552, 559), excision of lesion of chest wall (ET013) and insertion of prosthesis into chest wall NEC (ET023). If the patient received two of the listed curative operations in different episodes, the first operation was considered.

Cancer registry data include the date of diagnosis for each patient. The linkage of cancer records to HES allows the extract of all treatments that patients were recorded to have received between 1998 and 2006. It is possible that the treatment information in the HES extract is for a primary tumour prior to the one under study. For example, if a patient had a primary tumour in 1997 and a second primary in 2005, it is possible that the linkage process would result in analysing the treatment for the cancer in 1997 similar to that of the treatment for the cancer in 2005. To avoid this, only patients who underwent a surgical procedure between 1 month before and 6 months after cancer diagnosis were considered as treated by surgery.

Information on PCTs’ spending on cancer was extracted from the 2008–2009 Cancer Networks Workbook 1.1. This enables PCTs to compare spending on cancer within their geographic areas with that of other PCTs. Information on spending on lung cancer was collated from the NHS National Programme Budget Project (NPBP). This project aims to identify expenditure on 23 programmes of care including infectious diseases, cancer and mental health problems. Data on PCT spending by cancer site were available only for the years 2006/2007, 2007/2008 and 2008/2009, and show that spending on cancer varied for a number of PCTs from year to year.^26^ There is some indication that PCTs with low spending in 1 year tend to have increased spending in the following year.

### Statistical analysis

We fitted mixed effect logistic models to predict surgical treatment within 6 months after diagnosis (model 1) and mortality within 1 year of diagnosis (model 2). Multilevel models are designed for data grouped in clusters or hierarchies, with a single dependent variable at the lowest level and explanatory variables at each level of the hierarchy. With these models, we can evaluate how much of the variability of the dependent variable is attributed to the patients and how much is attributed to PCTs.

To better understand hierarchical models, let us assume that a total of n patients (level 1) are nested within J PCTs (level 2), with n_i_ patients in PCT_j_. By y_ij_, we denote the response of patient i in PCT_j_, where the response is either death or surgery (referred to respectively as models 1 and 2) (within the defined period). Assuming1
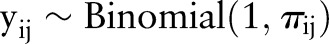
2
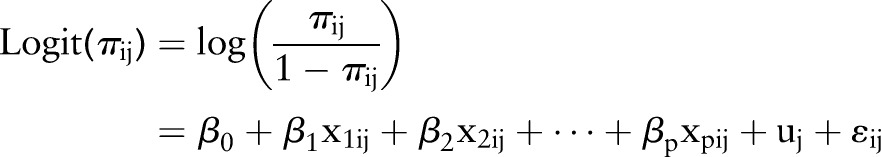
with 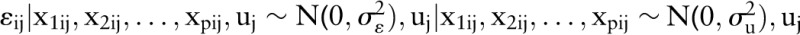 independent across PCTs and ε_ij_ are independent across both PCTs and patients, and x_kij_ representing the value of explanatory variable X_k_ for patient i in PCT j.^27^

The relationships between each of the two outcomes (on the log-odds scale) and two of the explanatory variables, age at diagnosis and year of diagnosis, are expected to be non-linear. For this reason, their effect is modelled using cubic splines, a set of piecewise polynomials of order 3 that are joined together to define a smooth curve.^28^ ^29^ The additional predictors in both models were sex and deprivation index. In these random intercept models, variations across PCTs are captured by the random component u_j_, which represents a PCT's departure from the mean log odds of PCTs.

To understand the impact of surgery on mortality within 1 year, model 2 was extended to include curative surgery within the window of 1 month before and up to 6 months after diagnosis (model 3).

We then examined whether the average amount spent on lung cancer could additionally explain the variation across PCTs. The average spending in pounds per 1000 patients for the three available years (2006/2007, 2007/2008 and 2008/2009) was included in model 2 as a proxy for spending during the full follow-up period (model 4). This average was grouped in four categories (<£3500, £3500–£4499, £4500–£5499, >£5000 thousand).

The predicted PCT-specific random intercepts derived from fitting the four mixed-effects models were used to visually identify PCTs with below-average curative surgery rates and those with above-average mortality from NSCLC, having accounted for the model-specific predictors using caterpillar plots. However, this approach does not adequately account for the variability of the estimates within each PCT. An alternative is to use funnel plots which have been advised for institutional comparisons of performance.^30^ Funnel plots have been widely used in meta-analysis to detect publication bias. They also became more popular recently in visualising performance indicators such as risk rates and relative survival estimates.^30–32^ Estimates are plotted with three superimposed lines, a ‘target’, or reference, quantity and upper and lower ‘control limits’, beyond which the estimate is considered an ‘outlier’. The control limits are calculated from a function of the statistical precision of the estimates. This gives the control limits the shape of the funnel with the wider part reflecting increased variability from less precise estimates. Funnel plots were therefore used to visually inspect the predicted PCT-specific random intercepts derived from each of the four mixed effect regression models, against their associated precision, given by the inverse of their variance. The target line was set at zero, because the expected value of the random intercept is zero 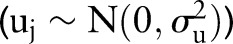, while the upper and lower limits were two SDs above and below this target line.

## Results

### Descriptive

A total of 228 247 patients were diagnosed with NSCLC during 1998–2006, and of these, 192 658 (84%) were identified within the extract of the HES database. The percentage of successful linkage with HES improved from 77% in 1998 to 86% in 2005. One record was excluded because of a missing postcode. A final data set of 192 657 records of patients diagnosed with NSCLC between 1998 and 2006 and followed up to 2010 and linked to HES episodes was analysed. The characteristics of these patients are detailed in table 1.

**Table 1:** Number (%) of patients with lung cancer by surgical treatment and mortality within 1 year

	N	Patients treated by surgery		Death within 1 year	
		N	Per cent	N	Per cent
All patients	**192 657**	**19 153**	**9**.**94**	**142 023**	**73.72**
Year of diagnosis					
1998	18 887	1883	9.97	14 244	75.42
1999	20 541	1961	9.55	15 589	75.89
2000	20 981	2133	10.17	15 502	73.89
2001	21 527	2203	10.23	15 922	73.96
2002	21 595	2168	10.04	15 846	73.38
2003	21 527	2138	9.93	15 749	73.16
2004	22 168	2091	9.43	16 162	72.91
2005	22 526	2232	9.91	16 311	72.41
2006	22 905	2344	10.23	16 698	72.90
Age (years)					
15–44	2540	515	20.28	1450	57.09
45–54	11 935	1855	15.54	7725	64.73
55–64	35 512	5358	15.09	23 533	66.27
65–74	64 784	7825	12.08	46 399	71.62
75–99	77 886	3600	4.62	62 916	80.78
Deprivation					
Most affluent	25 013	2694	10.77	17 950	71.76
2	31 796	3279	10.31	23 370	73.50
3	37 473	3772	10.07	27 501	73.39
4	46 605	4450	9.55	34 647	74.34
Most deprived	51 770	4958	9.58	38 555	74.47
Sex					
Male	117 966	11 601	9.83	87 865	74.48
Female	74 691	7552	10.11	54 158	72.51

Over 40% of the patients were older than 75 years of age; 61% were men and 26% were from the most deprived socioeconomic category. The unadjusted rate of death within the first year after diagnosis decreased slightly throughout the study period from 75% in 1998 to 73% in 2006; it was slightly higher among men and highest in the oldest age group (81%). Around 10% of the patients received curative surgery within the time window of 1 month before and up to 6 months after being diagnosed with cancer, and this percentage was almost unchanged throughout the study period.

The results of fitting the random intercept models for the odds of curative surgery and odds of 1-year mortality are reported in terms of ORs (table 2).

**Table 2:** Hierarchical random intercept logistic regression models with curative surgery (model 1) and mortality (model 2, model 3, model 4) as an outcome, random effects for PCTs adjusted for age, year of diagnosis, sex and deprivation

	Model 1			Model 2			Model 3			Model 4		
	OR	95% CI		OR	95% CI		OR	95% CI		OR	95% CI	
*Level 1: patients*												
Fixed effects												
Age at diagnosis												
71	1.00			1.00			1.00			1.00		
20	3.30	2.61	4.17	0.37	0.31	0.45	0.49	0.40	0.61	0.37	0.31	0.45
30	2.41	2.09	2.79	0.46	0.41	0.52	0.55	0.48	0.53	0.46	0.41	0.52
40	1.81	1.68	1.96	0.56	0.53	0.59	0.62	0.58	0.66	0.56	0.53	0.59
50	1.43	1.36	1.50	0.68	0.65	0.70	0.71	0.68	0.73	0.68	0.65	0.70
60	1.17	1.13	1.21	0.81	0.79	0.83	0.82	0.80	0.84	0.81	0.79	0.83
80	0.34	0.32	0.36	1.60	1.55	1.65	1.29	1.25	1.33	1.60	1.55	1.65
Year of diagnosis												
1998	1.00			1.00			1.00			1.00		
1999	1.04	1.02	1.07	0.96	0.94	0.98	0.96	0.95	0.98	0.96	0.94	0.98
2000	1.08	1.03	1.13	0.92	0.89	0.95	0.93	0.90	0.96	0.92	0.89	0.95
2001	1.09	1.03	1.16	0.89	0.85	0.92	0.89	0.86	0.93	0.89	0.85	0.92
2002	1.09	1.03	1.15	0.86	0.83	0.89	0.86	0.83	0.90	0.86	0.83	0.89
2003	1.07	1.02	1.12	0.84	0.81	0.87	0.83	0.80	0.86	0.84	0.81	0.87
2004	1.06	1.01	1.12	0.83	0.80	0.86	0.82	0.79	0.85	0.83	0.80	0.86
2005	1.09	1.04	1.14	0.83	0.80	0.85	0.82	0.79	0.85	0.83	0.80	0.85
2006	1.14	1.07	1.21	0.83	0.79	0.86	0.83	0.79	0.87	0.83	0.79	0.85
Sex												
Male	1.00			1.00			1.00			1.00		
Female	1.06	1.02	1.09	0.89	0.87	0.91	0.89	0.87	0.91	0.89	0.87	0.91
Deprivation												
Most affluent	1.00			1.00			1.00			1.00		
2	0.94	0.89	0.99	1.08	1.04	1.13	1.08	1.04	1.12	1.08	1.04	1.13
3	0.91	0.87	0.97	1.08	1.04	1.12	1.06	1.02	1.11	1.08	1.04	1.12
4	0.83	0.78	0.87	1.15	1.11	1.19	1.12	1.07	1.16	1.15	1.11	1.20
Most deprived	0.73	0.69	0.77	1.23	1.18	1.28	1.16	1.11	1.21	1.23		
Surgery												
No							1.00					
Yes							0.07	0.07	0.08			
Spending on lung cancer in pounds per 1000 patients												
<£3500										1.00		
£3500–£4499										0.93	0.87	0.99
£4500–£5499										0.95	0.89	1.01
>£5000										0.94	0.88	1.00
Intercept	0.08	0.08	0.09	3.04	2.90	3.18	4.22	4.01	4.43	3.18	3.00	3.37
*Level 2: PCTs*												
Random effect												
Variance	0.09	0.07	0.12	0.02	0.01	0.02	0.02	0.01	0.02	0.02	0.01	0.02

Model 1: Outcome—surgery within 1 month before and 6 months after diagnosis adjusted for covariates.

Model 2: Outcome—mortality within 1 year after diagnosis adjusted for covariates.

Model 3: Model 2, adjusted for surgery (patient level).

Model 4: Model 2, adjusted for spending on lung cancer (PCT level) per 1000 patients.

PCT, Primary Care Trust.

### Surgery

**Figure 1:**
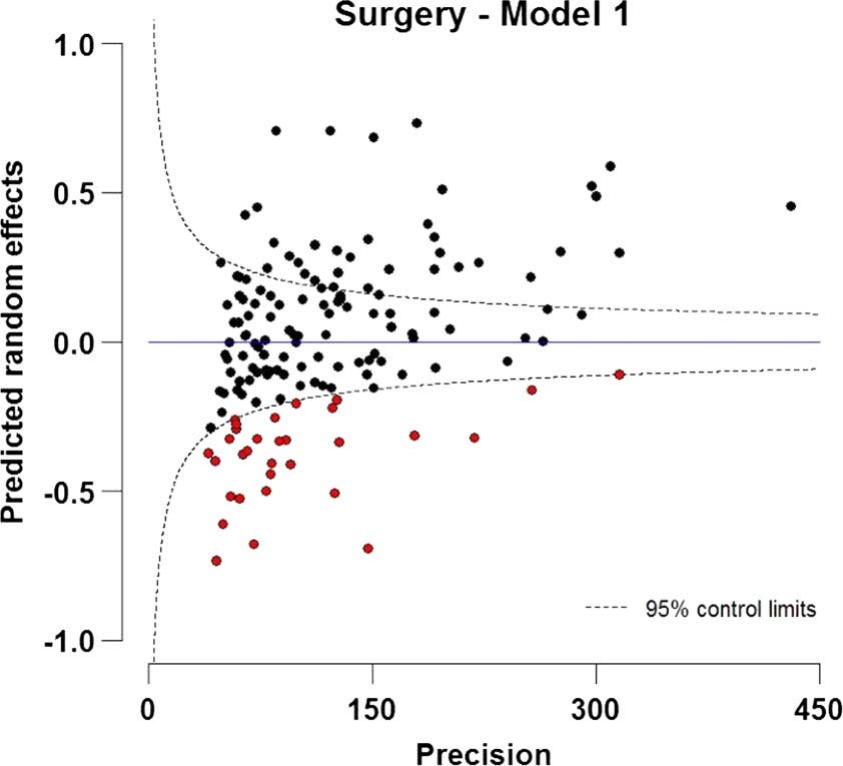
Funnel plot of predicted random intercepts of treatment by curative surgery within 1 year after diagnosis for patients with NSCLC (15–99 years) diagnosed during 1998–2006 in England. The target is fixed as zero, which specifies the expected value of the random effect.

A very strong and significant trend in the odds of surgery was found in relation to the deprivation index, with patients from deprived areas having a 27% lower likelihood of having surgery compared with affluent patients, controlling for sex, age and year of diagnosis (table 2). A clear trend by age group was seen in the proportion undergoing surgery. Younger patients were more likely to be treated by surgery, where the odds of having surgery were more than three times higher for those diagnosed at 20 years, and more than twice as high for patients diagnosed at the age of 30 years compared with patients diagnosed at the age of 71 years (mean age of diagnosis), again controlling for the other variables in the model (table 2). There was a very slight increase in the adjusted odds of surgery between 1998 and 2006. Figure 1 provides strong evidence of geographic differences in treatment by curative surgery across different PCTs. A total of 32 PCTs were below the lower control limit (indicated by red dots). The residual variance of the predicted PCT-specific random intercepts of surgery (model 1) was 0.09.

### Mortality within 1 year after diagnosis

Diagnosis of NSCLC at older ages is associated with higher odds of death, controlling for year of diagnosis, sex and deprivation index (table 2, model 2). Patients diagnosed at the age of 40 have almost half of the odds of death compared to those diagnosed at the age of 71 (mean age of diagnosis). Women experienced 11% lower odds of death in the first year after diagnosis compared to men, controlling for covariates. The results show a clear trend of the effect of deprivation index on mortality, with the most deprived patients having 23% higher odds of death compared to the most affluent patients, controlling for the other factors. The odds of death also declined slightly with later years of diagnosis. The residual variance of the predicted PCT-specific random intercepts was 0.02, smaller than that for surgery (model 2). A likelihood-ratio test comparing the model with a standard logistic regression model was highly significant (likelihood ratio test=1466.50; p<0.001).

The mixed effects model of mortality was further extended to control for curative surgery (model 3). The OR of death within the first year in patients treated by curative surgery was very small (0.07), relative to patients who did not undergo surgery, controlling for the other factors in the model (model 3). This implies that not receiving surgery is associated with more than 14 times the odds of death compared to those who receive surgery, controlling for covariates. However, the effects of age, year, sex and deprivation on mortality within 1 year barely changed after adjusting for surgery (model 3) (table 2, model 4).

**Figure 2:**
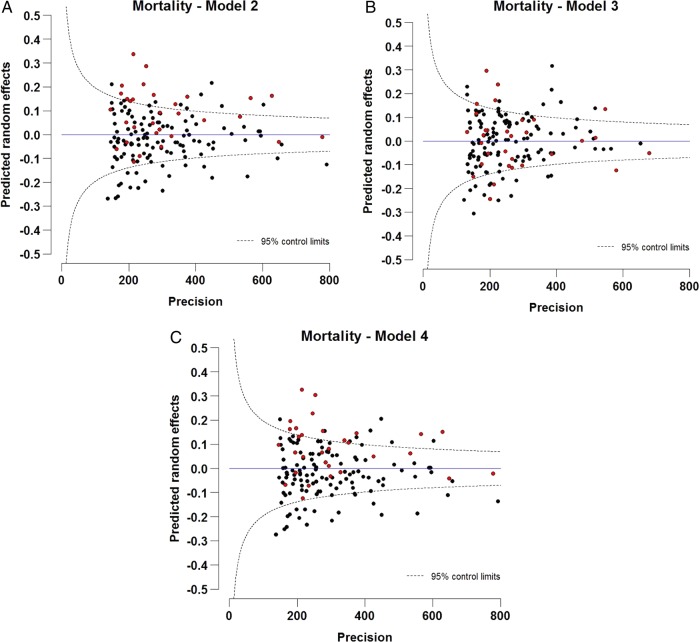
Funnel plots of predicted random intercepts of mortality within 1 year of diagnosis, controlling for (A) covariates (age, year of diagnosis, sex and deprivation) (model 2), (B) covariates and treatment by curative surgery (model 3), (C) covariates and spending on lung cancer at Primary Care Trust level (model 4) for patients with non-small cell lung cancer (15–99 years) diagnosed during 1998–2006 in England. The target is fixed as zero, which specifies the expected value of the random effect.

Figure 2 shows some evidence of geographical differences in the predicted PCT-specific random intercepts for mortality. PCTs identified in figure 1 as having a below-average uptake of surgery are identified in this figure in red. A total of 22 (69%) of 32 PCTs that were below the lower control limit of surgery have predicted random intercepts for mortality above zero, and 13 (40%) were above the upper control limit of mortality within 1 year (figure 2, model 2). Adjusting for surgery (model 3) explained some of this variation, highlighting the inverse association between undergoing surgery and mortality. Additionally, controlling for variation in spending across PCTs seemed to push some of the PCTs to have more extreme values (figure 2, model 4).

## Discussion

The results of this study indicate substantial geographical variation in the use of curative surgical resection and in mortality within 1 year for patients with NSCLC at the level of PCTs in England. These clear geographical disparities remained after adjusting for age at diagnosis, sex and socioeconomic deprivation.

Strong inverse relationships were found between the use of curative surgical treatment and both increasing age at diagnosis and deprivation, while short-term mortality also increased with increasing age and deprivation. This excess mortality reduced dramatically, in particular among the oldest group of patients after accounting for their lower proportion of surgical treatment. After adjusting for sociodemographic factors, the use of surgical treatment with curative intent increased regularly with a calendar year, while mortality decreased. However, at the national level, only a tenth of the patients diagnosed with NSCLC in 2006 received such surgical treatment.

Only one study investigated the variation in surgical treatment and mortality at the PCT level for the whole of England.^4^ They also found wide variation in surgical treatment between PCTs, and a strong inverse association between surgery treatment and mortality.^13^ However, these results did not account for the hierarchical structure of the data, that is, for the higher similarity (in particular for healthcare management) between patients within a given PCT than between those from different PCTs. We hypothesised that the PCTs that were below the lower control limit for surgery would be the ones with above-average mortality using funnel plots. Variation in mortality within the first year after diagnosis was also apparent by PCTs. Most of the PCTs that were below the lower limit for surgery were above average for mortality within 1 year, and 38% were above the upper control limit of mortality. We had clear evidence that surgical treatment explained some of the variation (model 3, figure 2) that was due to mortality after the adjustment of predictors.

The average rate of surgical treatment of the patients with NSCLC diagnosed during 1998–2006 in the linked records of cancer registry and the HES was almost 10%, which is lower than the reported rates in equally developed European countries, such as 24% for Italy^33^ and 18% for Sweden.^34^ The reasons for this are likely to be multifactorial and include a lower number of specialist thoracic surgeons^35^ and higher rates of comorbidities^33^ in the UK. Delays from first referral for diagnosis to assessment for surgery have also been suggested to be a factor.^36^

A clear trend in rates across deprivation categories was identified, where patients resident in deprived areas were less likely to undergo curative surgery, and more likely to die within 1 year after diagnosis. Our findings have already been supported by similar studies on the impact of age on treatment and mortality for patients with NSCLC^7^ and socioeconomic deprivation.^37–39^

PCTs are responsible for the management, planning and funding of healthcare in a small defined geographical area and therefore management of hospitals and treatment provided in their local territories. Until 2013, PCTs were responsible for 80% of the total NHS budget. We used hierarchical models to evaluate how much of the variability of the surgical treatment and mortality was attributed to the patients, and how much was attributed to PCTs, after adjusting for known predictors. We expect that much of the unexplained variability of mortality would be due to factors at the PCT level. The NHS implemented the NPBP in 2002 to monitor where NHS resources are invested. PCTs were asked to declare the proportion of their budget spent on major diseases including cancer. Information on overall spending on cancer at the PCT level was available only for 3 years from the NPBP. The average spending of the available 3 years was considered as an indication of spending over all the periods considered in this paper. The variability in mortality 1 year after diagnosis was not explained by the spending on lung cancer at the PCT level after controlling for other covariates at the patient level such as age, sex, deprivation and year of diagnosis. However, NPBP data have many limitations. The large variation in spending between PCTs suggests that the recorded spending of a considerable number of PCTs might have been inaccurate, especially in the early years when this programme was first implemented.^26^

The main strength of this study is the large national population-based cancer registry data analysis, which comprises complete cancer registration linked to high-quality information on treatment extracted from HES. The analysis accounted for the hierarchical structure of the data, with patients at the first level and PCTs at the second level. The mixed-effects models account for clustering and take into account the dependence of outcomes within a cluster, the PCT in our case. We show that funnel plots originally used for the comparisons of hospital-based estimates^30^ could also be used to identify outliers of random effects predicted by mixed effect models. This latter use helps to identify geographical variation in measures such as mortality and surgical treatment in population-based cancer data.

The major limitation, however, is the absence of information on well-known strong predictors of surgical treatment and mortality at the PCT level, such as stage at diagnosis, comorbidities,^40^ specialisation of the surgeon^41^ ^42^ and hospital volumes.^43^ Characteristics of the hospital at which the patient underwent surgery play an important role in the patients’ well-being after surgery. The patients with NSCLC first seen in a thoracic surgical centre were more likely to have surgery than those seen in non-surgical centres,^44^ and better survival outcomes have been demonstrated for centres with higher volumes of surgical procedures.^45^ We do not expect much change in spending on cancer allocated by PCTs within a range of 10 years;^26^ however, a more accurate measure on spending for the equivalent year of the cancer diagnosis of patients included in the study would have led to more accurate results.

Our main results suggest the presence of clear variation in the use of surgical treatment of NSCLC and mortality. Mixed-effects models combined with the funnel plot approach were useful for assessing disparities and assessing the PCTs that were above average in mortality and below average in surgery. It is clear that patients managed by PCTs with lower rates of surgical resection experience higher rates of mortality within 1 year after the diagnosis of NSCLC. To explain the variations in treatment and mortality that we have demonstrated, a range of other factors will need to be analysed in future studies, including geographical differences in waiting times from referral to surgery, stage at diagnosis, access to other non-surgical treatments and the nature of local multidisciplinary teams—especially the level of involvement of specialist thoracic surgeons. The configuration of the NHS in England is changing rapidly at the time of writing, and our work accordingly will need to be updated shortly. More accurate data on costs and expenditure might be expected from this current reorganisation, which may make it possible to more accurately examine the relationship between expenditure and outcomes in this common disease.

**What is already known on this subject?**

- Non-small cell lung cancer comprises approximately 85% of all lung cancer cases, and surgery is the preferred treatment for patients. The National Health Service established Primary Care Trusts (PCTs) in 2002 to manage local health needs.

**What this study adds?**

- Older deprived patients and those who did not receive surgery had much higher odds of death 1 year after being diagnosed with lung cancer. Mixed-effects models combined with the funnel plot approach were useful for assessing the performance of PCTs that were above average in mortality and below average in surgery.
